# Beverage Intake, Smoking Behavior, and Alcohol Consumption in Contemporary China—A Cross-Sectional Analysis from the 2011 China Health and Nutrition Survey

**DOI:** 10.3390/ijerph14050493

**Published:** 2017-05-07

**Authors:** Yen-Han Lee, Zhi Wang, Timothy C. Chiang, Ching-Ti Liu

**Affiliations:** 1 School of Public Health, Department of Applied Health Sciences, Indiana University, Bloomington, IN 47405, USA; zw34@indiana.edu; 2 College of Medicine, Pennsylvania State University, Hershey, PA 17033, USA; tchiang@hmc.psu.edu; 3 School of Public Health, Department of Biostatistics, Boston University, Boston, MA 02118, USA; ctliu@bu.edu

**Keywords:** China, beverage intake, smoking, alcohol consumption, Westernization, health behavior

## Abstract

Chinese residents enjoy various types of beverages in their daily life. With the rapid Westernization of contemporary China, several adverse health concerns—such as diabetes linked to sweetened beverages—have emerged. Until now, no research that examines associations between beverage consumption and smoking/drinking behaviors has been made available, despite the large Chinese populations partaking in such activities. We conducted a cross-sectional study to explore the association between beverage intake frequencies and smoking/drinking behaviors in 12,634 adult respondents who participated in the latest wave (2011) of the China Health and Nutrition Survey (CHNS). Further, we applied Tukey’s Honest Significance test for pairwise comparisons. We defined the consumption categories as daily (at least one serving per day), weekly (less than one serving per day, at least one serving per week), monthly (less than one serving per week, at least one serving per month), and less than monthly or none—for sweetened beverage, water, tea, and coffee consumptions. The data showed that both tea and sweetened beverages are associated with smoking/drinking behaviors. Compared to respondents who consume tea and sweetened beverages daily, the odds of smoking behaviors are lower for those who consume such beverages less frequently. Further policy implications are discussed, including higher taxes on sweetened beverages and lessons from other countries.

## 1. Introduction

China, with its citizens embracing changes in diet and activity patterns due in part to rapid Westernization since the early 21st century, has displayed a rapid increase in non-communicable diseases (NCDs), such as diabetes [1]. Between 2000 and 2010, the sale of carbonated beverages increased significantly in China, with the daily per capita sales of PepsiCo increasing by 127% and Coca-Cola increasing by 145% [2]. Large companies generate higher sales and revenues of the health of people all over the world. The global consumption of soft drinks increased from 1997 to 2010 from 9.5 gallons to 11.4 gallons per person per year; an additional 4.8 obese adults per 100 was associated with each 1% consumption increase [3]. The rise in sweetened beverage consumption globally has contributed to the obesity epidemic, increasing the prevalence of diabetes and cardiovascular diseases [4]. Given that the sweetened beverage industry has become a lucrative field, it is not surprising that these companies dedicate effort to targeting some of the world’s largest economies, including China. 

Still, China has a rich culture and history in other beverages, most notably: tea, various tea leaves, and tea-related products. The various tea options generate a large tea consumer population in China. Many positive effects arise from daily consumption. For example, a study of Chinese elderly people found daily and occasional tea drinkers had higher baseline verbal fluency than non-tea drinkers [5]. Other positive health effects of long-term green tea consumption include anti-obesity, anti-diabetic, and anti-viral activities [6]. Further, green tea contains compounds that downregulate pathways involved in cancer cell invasion as well [7]. 

Not all beverages have such positive effects. Coffee, which comprises a large part of the global beverage market, exhibits both harmful and beneficial effects. It has been associated with a decrease in chronic disease, such as type 2 diabetes [8], but its harmful associations include a higher incidence of cerebral infarction [9], and its withdrawal symptoms include fatigue and headache [10]. 

With various types of beverage intake, substance consumptions have also become major concerns in China, including alcohol consumption and smoking behaviors. Alcohol has long been a symbol of celebration and festivity, and is a catalyst for social and romantic relations [11]. By comparison, the issues related to alcohol use, such as chronic disease, accidents, and violence, have been largely neglected by the Chinese central government [12]. Behind alcohol, the other commonly used substance in China is tobacco [13]. The Chinese tobacco market is enormous, accounting for one third of the world’s smokers [14]. It is an activity in which an increasing amount of young people have been engaging as well, and by 2025, it is expected to contribute to 2 million deaths per year [15]. Even passive smokers are being affected; in 2002, it was estimated that lung cancer that arose from passive smoking resulted in a loss of nearly 230,000 healthy years—a measurement to define an individual’s years of life in good health condition [16]. Furthermore, a study found that approximately 36% of physicians smoke in the Shandong area, and smoking by healthcare professionals certainly makes combatting the tobacco problem more challenging [17]. 

As such, the consumption pattern of sweetened beverages and caffeine by some individuals can be worrisome. Although sweetened beverages have been found to cause chronic diseases [1], they remain popular in China. Between 2000 and 2010, the sales of Coca-Cola and PepsiCo soft drink products increased 149% and 129% in China, respectively [2]. Further, in contemporary China, it is very common to observe tea and sweetened beverage consumers smoking tobacco or drinking alcohol products; however, there is currently no scientific literature available to conclude such a claim. Smoking and alcohol consumption behaviors pose public health threats to Chinese consumers, but unhealthy beverage options could exacerbate consumers’ health conditions. There are growing health concerns for smoking and drinking behaviors alongside Westernized sweetened beverage options [1]. Research is needed to examine the associations between beverage intake frequencies (water, tea, sweetened beverages, and coffee) and smoking behaviors and alcohol consumption in contemporary China. Our study aims to fill this knowledge gap. Further policy implications will be discussed at the end of the manuscript.

## 2. Materials and Methods

### 2.1. Study Sample—China Health and Nutrition Survey (CHNS)

The investigators of this present study analyzed individual level datasets from the China Health and Nutrition Survey (CHNS). CHNS is a currently ongoing data of University of North Carolina-Chapel Hill and Chinese Center for Disease Control and Prevention (CCDC). This publicly available longitudinal data applies a multistage and random cluster procedure for data collection with goals to investigate the influence of health, nutrition, healthcare systems, and policies implemented by government agencies. The original wave was started in 1989, and subsequent waves were conducted every two, three, or four years; the most recent survey was conducted in 2011. Nine provinces were surveyed in the data collection process, including Liaoning, Heilongjiang, Jiangsu, Shandong, Henan, Hubei, Hunan, Guangxi, and Guizhou. Beijing, Chongqing, and Shanghai cities were newly added in the latest 2011 wave. From 1989 to 2011, the overall response rate relative to the previous round of survey is between 80 and 88 percent [18]. Further information can be found from the CHNS official website (http://www.cpc.unc.edu/projects/china), or the paper by Zhang et al. [18]. We applied only the latest individual level dataset for this research (2011), which was exempted from the approval of the Institutional Review Board (IRB) of University of North Carolina-Chapel Hill and CCDC based on its guidelines regarding individual level dataset from the CHNS. 

This study sample only includes adult respondents (age ≥ 18). We applied a cross-sectional design for analysis. The original study sample included 19,721 observations. We excluded subjects without full responses to questions of interests. After we removed missing values, a total of 12,634 individuals were retained for the final study sample (n = 12,634).

### 2.2. Outcome Variables

We considered three outcome variables to define smoking and drinking consumptions: (1) smoking experience regarding whether the individual had ever smoked in the past (No–Yes, dichotomous variable); (2) current smoking status (No–Yes, dichotomous variable); and (3) alcohol consumption in the past year (No–Yes, dichotomous variable). 

### 2.3. Predictors

There are four predictors of beverage intake frequencies (categorical variables): sweetened beverages, water, tea, and coffee. According to the questionnaire, the coding was based on a 24 h dietary recall and self-reported intake frequency on the CHNS. We classified the consumption categories for all predictors into “daily” (at least one serving per day), “weekly” (less than one serving per day, at least one serving per week), “monthly” (less than one serving per week, at least one serving per month), and less than monthly or none. 

### 2.4. Covariates

We accounted for the following covariates in our statistical models: age (in years, continuous variable), gender (Male–Female, dichotomous variable), level of education (categorical variable), employment status (No–Yes, dichotomous variable), marital status (Not married–married, dichotomous variable), region (Rural–Urban, dichotomous variable), and province (categorical variable). These variables were selected to serve as surrogated indicators for respondents’ regional information, socioeconomic status, and basic biological information. 

We regrouped categories with similar characteristics. In case of education level, our classifications include “lower” (no education and elementary school education), “middle school” (lower middle school and upper middle school education), and “higher education” (university/college and above). We kept vocational/technical school education in its original format. Provincial categories include “North East” (Liaoning and Heilongjiang provinces), “East Coastal” (Jiangsu and Shandong provinces), “Central” (Henan and Hubei provinces), “South” (Hunan, Guangxi, and Guizhou provinces), and “Municipals” (Beijing, Chongqing, and Shanghai). 

### 2.5. Statistical Analysis

We examined the crude associations between outcome and independent variable with chi-square tests for categorical predictors and a Student’s *t*-test for continuous predictor (e.g., age), and conducted multivariable logistic regression models for all statistical analyses. Further, we performed Tukey’s Honest Significance tests to examine the associations between each beverage intake frequency. All statistical analyses were performed on the statistical package R, version 3.3.1.

## 3. Results

### 3.1. Sample Characteristics 

We analyzed 12,634 individuals (52.9% females), with a mean age of 51.1 ± 15 years (Table 1). Most respondents were married and employed, but more than 80% of the study sample only received a high school education or lower. More than 50% of individuals resided in southern provinces and municipals. Approximately 90% of the respondents consumed water, but only 6% of the study subjects drank coffee (Table 1). Compared with other beverage intake frequencies, approximately 80% of participants consumed water daily. As for smoking behaviors, nearly 31% of respondents had smoking experiences, and 26% of them were current smokers. Alcohol consumption behavior in the past year was around 34% (Table 1). Daily tea drinkers had higher prevalence rates on smoking and past year alcohol consumption behaviors, compared to individuals with daily sweetened beverage and coffee consumptions (Table 1). 

### 3.2. Associations of Beverage Intake Frequencies with Smoking Behaviors and Alcohol Consumption

For beverage intake frequencies, sweetened beverage frequency is associated with smoking behaviors. Non-daily consumers of sweetened beverages have lower odds of being smokers than those with daily consumption. Similar patterns, except in monthly consumption, were observed in tea intake frequencies with smoking behaviors (Table 2). Weekly tea consumption is associated with past year alcohol consumption, and has higher odds (OR: 1.19, 95% CI = 1.02–1.39), compared with daily tea consumption. However, of the association with statistical significance, individuals with less than monthly tea consumption have less odds (OR: 0.53, 95% CI = 0.47–0.59) to consume alcohol in the past year, compared with those who drank on a daily basis. In terms of water intake, most drinking frequencies are not associated with any smoking behaviors and alcohol consumption, with the exception of the weak associations between less than monthly or none intake and past year alcohol consumption (OR: 0.85, 95% CI = 0.73–0.99). 

Table 3 provides comprehensive pairwise comparison results. Smoking behaviors are associated with sweetened beverage consumption (*p* < 0.05). Specifically, smoking experiences are statistically significant associated with sweetened beverage consumption frequencies between weekly and daily consumption, monthly and daily consumption, and less than monthly (or none) and daily consumption (Table 3). However, as for current smoking status, weekly and daily comparison does not have statistically significant associations (*p* = 0.055). In terms of past year alcohol consumption, sweetened beverage consumption frequency only demonstrates statistically significant association between monthly and weekly (*p* = 0.012). The pairwise comparisons for tea consumption frequency between weekly and daily consumption, less than monthly and daily consumption, and less than monthly and weekly consumption are associated with smoking behaviors. Furthermore, all comparisons with less than monthly tea consumption or none have statistically significant associations with past year alcohol consumption (Table 3). 

## 4. Discussion

To the best of the authors’ knowledge, this is the first investigation of the direct associations between beverage intake frequencies and smoking/drinking consumption behaviors in contemporary China. In our research, tea and sweetened beverage consumptions were significantly associated with smoking statuses and past year alcohol consumption behavior (*p* < 0.05), but water and coffee intakes were not associated with any outcomes based on overall *p*-values (Table 2 and Table 3). First, the possibly alternative explanation for observation on water is that water has been considered significant nutrients to human beings [19], and people need to rely on water sources to maintain physiological function, regardless of their smoking status and alcohol consumption. 

In general, respondents, who consumed tea daily, were more likely to have smoking experiences, become current smokers, and have alcohol consumption in the past year, compared with people who did not drink tea on a daily basis (Table 2), although the health benefits of tea consumption have been studied previously [6,7]. The tea consumers largely overlap with populations of smoking and alcohol consumption in China (Table 1), given that the cigarettes, alcohol, and tea products have represented personal habits in everyday life [20]. Tea as a gift option has become more popular, even though there is currently no formal or direct statistics to support this. In China, tea and other gifts, including cigarettes and alcoholic beverages, are used to build “Guanxi,” a special relationship with favor and debt, and to link between host and guest. This phenomenon is common in Chinese culture, even in old feudalistic society. For instance, tea was once used to represent a connection between superior and subordinate in the Qing dynasty [21]. Tea consumers receiving gifts usually have social economic advantages. Such populations are also more likely to smoke and drink alcohol. Future interventions should focus on populations with similar behaviors. For example, public health endeavors should target populations by modifying the smoking and drinking behaviors of tea consumers. Additionally, practitioners should educate audiences regarding the health benefits of tea consumption, and separate tea products from harmful behaviors, such as smoking and drinking.

Coffee drinking is less popular in China, although coffee shops are accessible to most residents, especially for the convenient accessibility in metropolitan areas. For instance, Starbucks, the popular global coffee chain store from the United States, has plans to expand its market and launch nearly 5000 stores by 2021 in China [22,23]. However, only 5.3% of respondents consumed coffee at least monthly based on our data (Table 1). Even though China is emerging as a potential roasted coffee consumption market, the primary coffee consumption is still instant coffee, also known as a “3-in-1” coffee beverage in China [24], a product that contains instant coffee, sugar, and cream powder. Overall coffee consumption has been associated with a lower rate of total mortality in the United States and a lower risk of type 2 diabetes [8,25]. However, our concern is that the “3-in-1” product could be a confounding factor in coffee- and health-related research, regardless of caffeinated or decaffeinated instant coffee products. Further research to study such associations with the distinction of instant coffee products is warranted. 

Overall frequency of sweetened beverage consumption is strongly associated with smoking behaviors. Among subjects with smoking experiences, sweetened beverage consumption has positive associations with such history. Respondents with less sweetened beverage consumptions were less likely to smoke currently or to have smoking experiences (Table 2). First, our observations are consistent with previous studies that tobacco users are more likely to have other unhealthy dietary and health behaviors [26,27]. Sweetened beverage consumption as a predictor is associated with a series of unhealthy behaviors that impact consumer health status and chronic disease [28,29]. Furthermore, some addiction behaviors are able to cross different domains; for example, substance-dependent individuals may crave food with a high concentration of sucrose [30,31,32]. Such interactions between sweetened beverage consumption and smoking behaviors should be considered together in order to maximize the effects of public health policy in the future. Meanwhile, there has been a global trend of taxing sweetened beverages in recent years in order to reduce sweetened beverage consumption and promote public health, for example, in the United Kingdom [33] and some places in the United States [34]. Research has shown that taxes on sweetened beverages help to reduce obesity and sweetened beverage consumption in the United States [34]. However, no such action or data has been observed regarding sweetened beverages in China. Further studies are needed to investigate the necessity of such efforts in China with more comprehensive datasets. Chinese government agencies should consider taking further action to increase taxes on sweetened beverages in order to decrease consumption in the near future. A similar taxation plan should also be applied to tobacco products. Indeed, China has made adjustments in the last few years to increase taxes on tobacco products; the results have shown a slight decline from 2014 to 2015 by approximately 2.4% for total cigarette sales [35]. Even though these numbers are not large, the future for public health promotion remains optimistic in China, and there is potential for further changes in health behavior.

The Chinese central government has noticed public health issues (such as poor eating habits and lifestyle), and has planned to implement further interventions to reduce the prevalence of poor habits [36,37]. Chinese residents’ health behaviors are largely influenced by Westernization, including poor eating habits, binge drinking, and continuing smoking behavior. This rapid Westernization, especially in large cities, may thwart further health strategic interventions. Without effective prevention and health promotion, it is expected that the cancer rate will be doubled or tripled in the next two decades due to poor lifestyle [38]. Stakeholders should continue to cooperate with government agencies to achieve the goal of a healthier living environment. 

There are also some caveats for this present study. First, we do not have information from CHNS to examine whether alcohol consumers identify themselves as “past alcohol users” or “current users”. Further research should address this issue to investigate associations with alcohol consumption in the long term, rather than just past year consumption. Secondly, the questionnaire measurements may not reflect actual beverage intake behaviors among respondents. For example, based on our data, we observed that nearly 11 percent of total respondents drank water less than once a month or did not drink water at all (Table 1). Further, the 24 h dietary recall method from the CHNS cannot correctly capture respondents’ long-term beverage intake. Other rigorous data collection strategies regarding dietary intake should be considered by others. Third, in terms of coffee consumption, we did not have information to differentiate the caffeinated coffee, decaffeinated coffee, or “3-in-1” coffee products; different coffee types may be a confounding factor. This limitation prevents us from making a conclusive claim on coffee consumption.

## 5. Conclusions

In sum, this is the first study, to the best of the authors’ knowledge, to investigate the associations between beverage intake frequencies and smoking/drinking consumption behaviors in contemporary China. We observed that consumers who did not frequently consume sweetened beverages and tea products had less odds to have smoking experience, or become current smokers, compared with those who consumed daily. The Chinese central government should continue to promote healthy dietary behaviors, including healthy beverage intakes, to prevent increasing prevalence of non-communicable diseases, smoking behaviors, and alcohol uses in China. Researchers and policy makers should investigate similar topic of interest further. 

## Figures and Tables

**Table 1:** Descriptive statistics, prevalence rates by variables, and crude examinations for study sample (n = 12,634). Data source: China Health and Nutrition Survey, 2011.

Variables	Observations (N = 12,634)	Smoking Experience (3873 Yes, 30.7%)	Current Smoking Status (3328 Yes, 26.3%)	Past Year Alcohol Consumption (4279 Yes, 33.9%)
		*p*-value ^a^	*p*-value ^d^	*p*-value ^g^
	N_1_ (%)	N_2 _^b^ (pre.smk ^c^)	N_3 _^e^ (pre.csmk ^f^)	N_4 _^h^ (pre.alc ^i^)
Primary predictors:				
**Frequency: sweetened beverage consumption**		<0.001	<0.001	0.042
Daily	214 (1.7)	96 (0.8)	82 (0.6)	88 (0.7)
Weekly	1546 (12.2)	436 (3.5)	395 (3.1)	547 (4.3)
Monthly	1670 (13.2)	407 (3.2)	349 (2.8)	577 (4.6)
Less than monthly or none	9204 (72.9)	2934 (23.2)	2502 (19.8)	3067 (24.3)
**Frequency: water consumption**		<0.001	<0.001	<0.001
Daily	10539 (83.4)	3013 (23.8)	2579 (20.4)	3442 (27.2)
Weekly	660 (5.2)	222 (1.8)	193 (1.5)	239 (1.9)
Monthly	64 (0.5)	21 (0.2)	19 (0.2)	23 (0.2)
Less than monthly or none	1371 (10.9)	617 (4.9)	537 (4.3)	575 (4.6)
**Frequency: tea consumption**		<0.001	<0.001	<0.001
Daily	3499 (27.7)	1625 (12.9)	1405 (11.1)	1664 (13.2)
Weekly	1325 (10.5)	441 (3.5)	376 (3.0)	626 (5.0)
Monthly	117 (0.9)	42 (0.3)	32 (0.3)	49 (0.4)
Less than monthly or none	7693 (60.9)	1765 (14.0)	1515 (12.0)	1940 (15.4)
**Frequency: coffee consumption**		0.225	0.495	<0.001
Daily	143 (1.1)	43 (0.3)	33 (0.3)	54 (0.4)
Weekly	387 (3.1)	101 (0.8)	93 (0.7)	174 (1.4)
Monthly	138 (1.1)	39 (0.3)	33 (0.3)	64 (0.5)
Less than monthly or none	11966 (94.7)	3690 (29.2)	3169 (25.1)	3987 (31.6)
Covariates:				
**Age** **(mean ± SD)**	51.1 ± 15.3	<0.001	0.615	<0.001
**Gender**		<0.001	<0.001	<0.001
Male	5946 (47.1)	3643 (28.8)	3134 (24.8)	3524 (27.9)
Female	6688 (52.9)	230 (1.8)	194 (1.5)	755 (6.0)
**Education level**		<0.001	<0.001	<0.001
Lower	4530 (35.9)	1303 (10.3)	1090 (8.6)	1133 (9.0)
Middle school	5628 (44.5)	1930 (15.3)	1702 (13.5)	2111 (16.7)
Vocational	925 (7.3)	272 (2.2)	228 (1.8)	360 (2.8)
Higher	1551 (12.3)	368 (2.9)	308 (2.4)	675 (5.3)
**Employment status**		<0.001	<0.001	<0.001
No	5345 (42.3)	1261 (10.0)	959 (7.6)	1244 (9.8)
Yes	7289 (57.7)	2612 (20.7)	2369 (18.8)	3035 (24.0)
**Marital status**		<0.001	<0.001	<0.001
No	2000 (15.8)	485 (3.8)	424 (3.4)	513 (4.1)
Yes	10634 (84.2)	3388 (26.8)	2904 (23.0)	3766 (29.8)
**Rural/urban communities**		<0.001	<0.001	0.576
Rural	7307 (57.8)	2420 (19.2)	2123 (16.8)	2490 (19.7)
Urban	5327 (42.2)	1453 (11.5)	1205 (9.5)	1789 (14.2)
**Provinces**		0.141	0.018	<0.001
North East	1901 (15.0)	606 (4.8)	520 (4.1)	599 (4.7)
East Coastal	2135 (16.9)	617 (4.9)	532 (4.2)	727 (5.8)
Central	2036 (16.1)	603 (4.8)	512 (4.1)	704 (5.6)
South	3280 (26.0)	1037 (8.2)	926 (7.3)	1018 (8.1)
Municipals	3282 (26.0)	1010 (8.0)	838 (6.6)	1231 (9.7)

Statistical significance: *p*-value <0.05. Prevalence rate: Number of cases/Number of total population: ^a^ *p*-value: the *p*-value of the crude association between smoking experience and predictor. ^b^ N_2_: Distribution of individuals with smoking experience by variable. ^c^ Pre.smk: Prevalence rate of individuals with smoking experience. ^d^ *p*-value: the *p*-value of the crude association between current smoking status and predictor. ^e^ N_3:_ Distribution of individuals with current smoking status by variable. ^f^ Pre.csmk: Prevalence rate of current smokers. ^g^ *p*-value: the *p*-value of the crude association between past year alcohol consumption and predictor. ^h^ N_4_: Distribution of individuals with past year alcohol consumption by variable. ^i^ Pre.alc: Prevalence of individuals with past year alcohol consumption.

**Table 2:** Odds ratios (ORs) and 95 percent confidence intervals (95% CI): Associations with beverage intake frequencies on smoking and alcohol consumption behaviors adjusted with all covariates. Data source: China Health and Nutrition Survey, 2011.

Variables	Smoking Experience	Current Smoking Status	Past Year Alcohol Consumption
	*OR, 95% CI*	*OR, 95% CI*	*OR, 95% CI*
Age	1.01 **, 1.01–1.02	1.00, 0.99–1.00	0.99 **, 0.99–1.00
Gender			
Female	0.02 **, 0.02–0.03	0.03 **, 0.02–0.03	0.10 **, 0.09–0.11
Education level			
(Overall *p*-value)	(<0.001)	(<0.001)	(<0.001)
Lower	1.00	1.00	1.00
Middle school	0.77 **, 0.68–0.88	0.79 **, 0.70–0.90	1.21 **, 1.08–1.35
Vocational	0.60 **, 0.48–0.74	0.58 **, 0.46–0.71	1.41 **, 1.16–1.70
Higher	0.35 **, 0.28–0.42	0.35 **, 0.28–0.42	1.47 **, 1.24–1.74
Employment status			
Yes	1.46 **, 1.29–1.65	1.70 **, 1.50–1.92	1.61 **, 1.45–1.79
Marital status			
Yes	1.02, 0.88–1.19	1.00, 0.86–1.16	1.29 **, 1.13–1.48
Rural/urban communities			
Urban	0.74 **, 0.66–0.83	0.77 **, 0.68–0.86	0.81 **, 0.72–0.90
Provinces			
(Overall *p*-value)	(<0.001)	(<0.001)	(<0.001)
North East	1.00	1.00	1.00
East Coastal	0.66 **, 0.55–0.79	0.71 **, 0.60–0.85	1.09, 0.93–1.27
Central	0.77 **, 0.64–0.92	0.77 **, 0.65–0.93	1.23 *, 1.05–1.44
South	0.82 *, 0.69–0.96	0.92, 0.78–1.08	0.89, 0.76–1.03
Municipals	0.99, 0.83–1.18	0.93, 0.78–1.11	1.23 **, 1.06–1.44
Sweetened beverage consumption			
(Overall *p*-value)	(<0.001)	(0.004)	(0.020)
Daily	1.00	1.00	1.00
Weekly	0.47 **, 0.31–0.69	0.62 *, 0.42–0.90	0.89, 0.63–1.25
Monthly	0.40 **, 0.27–0.60	0.51 **, 0.35–0.75	1.16, 0.82–1.64
Less than monthly or none	0.44 **, 0.30–0.64	0.61 **, 0.42–0.87	0.97, 0.70–1.35
Water consumption			
(Overall *p*-value)	(0.512)	(0.258)	(0.079)
Daily	1.00	1.00	1.00
Weekly	1.07, 0.85–1.34	1.08, 0.86–1.34	0.93, 0.76–1.13
Monthly	0.68, 0.35–1.30	0.82, 0.43–1.56	0.63, 0.34–1.13
Less than monthly or none	1.07, 0.90–1.26	1.16, 0.99–1.37	0.85 *, 0.73–0.99
Tea consumption			
(Overall *p*-value)	(<0.001)	(<0.001)	(<0.001)
Daily	1.00	1.00	1.00
Weekly	0.68 **, 0.57–0.81	0.68 **, 0.57–0.81	1.19 *, 1.02–1.39
Monthly	0.89, 0.53–1.52	0.68, 0.41–1.12	0.99, 0.63–1.54
Less than monthly or none	0.51 **, 0.45–0.58	0.54 **, 0.48–0.62	0.53 **, 0.47–0.59
Coffee consumption			
(Overall *p*-value)	(0.437)	(0.864)	(0.147)
Daily	1.00	1.00	1.00
Weekly	0.70, 0.40–1.24	0.99, 0.57–1.75	1.15, 0.72–1.84
Monthly	0.96, 0.48–1.93	1.11, 0.56–2.21	1.40, 0.80–2.46
Less than monthly or none	0.73, 0.45–1.20	0.93, 0.57–1.52	0.95, 0.63–1.42

* *p*-value < 0.05, ** *p*-value < 0.01; OR: 1.00 (Reference level).

**Table 3:** Pairwise comparisons between each beverage intake frequency from Tukey’s Honest Significance tests. Data source: China Health and Nutrition Survey, 2011.

Pairwise Comparisons	Smoke Experience	Current Smoking Status	Past Year Alcohol Consumption
*Sweetened beverage consumption:*			
Weekly—Daily	0.001	0.055	0.898
Monthly—Daily	<0.001	0.003	0.816
Less than monthly or none—Daily	<0.001	0.031	0.999
Monthly—Weekly	0.437	0.241	0.012
Less than monthly or none—Weekly	0.886	0.997	0.567
Less than monthly or none—Monthly	0.637	0.129	0.048
*Water consumption:*			
Weekly—Daily	0.932	0.902	0.852
Monthly—Daily	0.615	0.922	0.376
Less than monthly or none—Daily	0.858	0.231	0.127
Monthly—Weekly	0.526	0.842	0.571
Less than monthly or none—Weekly	1.000	0.927	0.879
Less than monthly or none—Monthly	0.503	0.696	0.726
*Tea consumption:*			
Weekly—Daily	<0.001	<0.001	0.113
Monthly—Daily	0.972	0.401	1.000
Less than monthly or none—Daily	<0.001	<0.001	<0.001
Monthly—Weekly	0.722	1.000	0.838
Less than monthly or none—Weekly	0.003	0.029	<0.001
Less than monthly or none—Monthly	0.124	0.810	0.022
*Coffee consumption:*			
Weekly—Daily	0.595	1.000	0.929
Monthly—Daily	0.999	0.990	0.627
Less than monthly or none—Daily	0.584	0.988	0.992
Monthly—Weekly	0.689	0.978	0.832
Less than monthly or none—Weekly	0.993	0.968	0.420
Less than monthly or none—Monthly	0.697	0.882	0.219

Statistical significance: *p* < 0.05.
