# Correction: Lee, Y.-H., et al. Beverage Intake, Smoking Behavior, and Alcohol Consumption in Contemporary China—A Cross-Sectional Analysis from the 2011 China Health and Nutrition Survey. *Int. J. Environ. Res. Public Health* 2017, *14*, 493

**DOI:** 10.3390/ijerph15050846

**Published:** 2018-04-25

**Authors:** Yen-Han Lee, Zhi Wang, Timothy C. Chiang, Ching-Ti Liu

**Affiliations:** 1 School of Public Health, Department of Applied Health Sciences, Indiana University, Bloomington, IN 47405, USA; zw34@indiana.edu; 2 College of Medicine, Pennsylvania State University, Hershey, PA 17033, USA; tchiang@hmc.psu.edu; 3 School of Public Health, Department of Biostatistics, Boston University, Boston, MA 02118, USA; ctliu@bu.edu

The authors wish to add the following amendments and corrections to their paper published in IJERPH [1].

1. All “consumptions” in the manuscript should be corrected as “consumption”.
2. Minor errors in statistical calculations have been observed. New statistical analyses have been performed. The newly conducted analyses do not change the conclusions of this study. We have attached the edited tables (Table 1, Table 2 and Table 3).
3. The results of Tukey’s honest significance tests were mostly similar, except with respect to the association between weekly and daily consumption of sweetened beverages and current smoking status. However, this new result is more consistent with that of the multivariable logistic regression model.

## Figures and Tables

**Table 1:** Descriptive statistics, prevalence rates by variables, and crude examinations for the study sample (N = 12,658). Data source: Chinese Health and Nutrition Survey, 2011.

Variables	Observations (N = 12,658)	Smoking Experience (3883 Yes, 30.7%)	Current Smoking Status (3337 Yes, 26.4%)	Past Year Alcohol Consumption (4289 Yes, 33.9%)
		^a^*p*-Value	^d^*p*-Value	^g^*p*-Value
	N_1_ (%)	^b^ N_2_ (^c^ pre.smk)	^e^ N_3_ (^f^ pre.csmk)	^h^ N_4_ (^i^ pre.alc)
Primary predictors:				
**Frequency:**				
**Sweetened beverage consumption**		<0.001	<0.001	0.038
Daily	215 (1.7)	97 (0.8)	83 (0.7)	89 (0.7)
Weekly	1548 (12.2)	437 (3.5)	395 (3.1)	547 (4.3)
Monthly	1673 (13.2)	408 (3.2)	350 (2.8)	578 (4.6)
Less than monthly or none	9222 (72.9)	2941 (23.2)	2509 (19.8)	3075 (24.3)
**Frequency:**				
**Water consumption**		<0.001	<0.001	<0.001
Daily	10,555 (83.4)	3019 (23.9)	2585 (20.4)	3450 (27.3)
Weekly	661 (5.2)	223 (1.8)	194 (1.5)	240 (1.9)
Monthly	64 (0.5)	21 (0.2)	19 (0.2)	23 (0.2)
Less than monthly or none	1378 (10.9)	620 (4.9)	539 (4.3)	576 (4.6)
**Frequency:**				
**Tea consumption**		<0.001	<0.001	<0.001
Daily	3507 (27.7)	1629 (12.9)	1408 (11.1)	1666 (13.2)
Weekly	1331 (10.5)	444 (3.5)	379 (3.0)	630 (5.0)
Monthly	117 (0.9)	42 (0.3)	32 (0.3)	49 (0.4)
Less than monthly or none	7703 (60.9)	1768 (14.0)	1518 (12.0)	1944 (15.4)
**Frequency:**				
**Coffee consumption**		0.209	0.476	<0.001
Daily	143 (1.1)	43 (0.3)	33 (0.3)	54 (0.4)
Weekly	388 (3.1)	101 (0.8)	93 (0.7)	175 (1.4)
Monthly	138 (1.1)	39 (0.3)	33 (0.3)	64 (0.5)
Less than monthly or none	11,989 (94.7)	3700 (29.2)	3178 (25.1)	3996 (31.6)
Covariates:				
**Age**				
**(mean ± SD)**	51.1 ± 15.3	<0.001	0.566	<0.001
**Gender**		<0.001	<0.001	<0.001
Male	5959 (47.1)	3652 (28.9)	3142 (24.8)	3530 (27.9)
Female	6699 (52.9)	231 (1.8)	195 (1.5)	759 (6.0)
**Education level**		<0.001	<0.001	<0.001
Lower	4538 (35.9)	1306 (10.3)	1093 (8.6)	1136 (9.0)
Middle school	5636 (44.5)	1934 (15.3)	1705 (13.5)	2114 (16.7)
Vocational	929 (7.3)	274 (2.2)	230 (1.8)	361 (2.9)
Higher	1555 (12.3)	369 (2.9)	309 (2.4)	678 (5.4)
**Employment status**		<0.001	<0.001	<0.001
No	5355 (42.3)	1266 (10.0)	963 (7.6)	1248 (9.9)
Yes	7303 (57.7)	2617 (20.7)	2374 (18.8)	3041 (24.0)
**Marital status**		<0.001	<0.001	<0.001
No	2003 (15.8)	487 (3.8)	426 (3.4)	514 (4.1)
Yes	10,655 (84.2)	3396 (26.8)	2911 (23.0)	3775 (29.8)
**Rural/urban communities**		<0.001	<0.001	0.571
Rural	7318 (57.8)	2424 (19.1)	2127 (16.8)	2495 (19.7)
Urban	5340 (42.2)	1459 (11.5)	1210 (9.6)	1794 (14.2)
**Provinces**		0.16	0.021	<0.001
Northeast	1902 (15.0)	606 (4.8)	520 (4.1)	600 (4.7)
East coast	2139 (16.9)	619 (4.9)	533 (4.2)	729 (5.8)
Central	2038 (16.1)	605 (4.8)	514 (4.1)	704 (5.6)
South	3291 (26.0)	1040 (8.2)	929 (7.3)	1022 (8.1)
Municipal	3288 (26.0)	1013 (8.0)	841 (6.6)	1234 (9.7)

Statistical significance: *p*-value < 0.05. Prevalence rate: Number of cases/Number of total population. ^a^ *p*-value: the *p*-value of the crude association between smoking experience and the predictor; ^b^ N_2_: Distribution of individuals with smoking experience by variable; ^c^ pre.smk: Prevalence rate of individuals with smoking experience; ^d^ *p*-value: the *p*-value of the crude association between current smoking status and the predictor; ^e^ N_3_: Distribution of individuals with current smoking status by variable; ^f^ pre.csmk: Prevalence rate of current smokers; ^g^ *p*-value: the *p*-value of the crude association between past year alcohol consumption and the predictor; ^h^ N_4_: Distribution of individuals with past year alcohol consumption by variable; ^i^ pre.alc: Prevalence of individuals with past year alcohol consumption.

**Table 2:** Odds ratios (OR) and 95 percent confidence intervals (95% CI): Associations among beverage intake frequencies, smoking, and alcohol consumption behaviors are adjusted for all covariates. Data source: China Health and Nutrition Survey, 2011.

Variables	Smoking Experience	Current Smoking Status	Past Year Alcohol Consumption
	OR 95% CI	OR 95% CI	OR 95% CI
**Age**	1.01 ** 1.01–1.02	1.00 0.99–1.00	0.99 ** 0.99–1.00
**Gender**			
Female	0.02 ** 0.02–0.03	0.03 ** 0.02–0.03	0.10 ** 0.09–0.11
**Education level**			
**(Overall *p*-value)**	(<0.001)	(<0.001)	(<0.001)
Lower	1	1	1
Middle school	0.77 ** 0.68–0.88	0.79 ** 0.70–0.90	1.21 ** 1.08–1.36
Vocational	0.60 ** 0.48–0.74	0.58 ** 0.46–0.71	1.40 ** 1.16–1.69
Higher	0.34 ** 0.28–0.42	0.35 ** 0.28–0.42	1.47 ** 1.24–1.75
**Employment status**			
Yes	1.45 ** 1.28–1.64	1.69 ** 1.49–1.90	1.61 ** 1.45–1.78
**Marital status**			
Yes	1.02 0.88–1.19	1.00 0.86–1.16	1.30 ** 1.14–1.48
**Rural/urban communities**			
Urban	0.74 ** 0.66–0.84	0.77 ** 0.68–0.86	0.80 ** 0.72–0.89
**Provinces**			
**(Overall *p*-value)**	(<0.001)	(<0.001)	(<0.001)
Northeast	1	1	1
East coast	0.66 ** 0.55–0.79	0.71 ** 0.60–0.85	1.09 0.93–1.28
Central	0.77 ** 0.64–0.92	0.78 ** 0.65–0.93	1.22 * 1.04–1.44
South	0.81 * 0.69–0.96	0.92 0.78–1.08	0.89 0.76–1.03
Municipal	0.99 0.83–1.18	0.94 0.79–1.11	1.23 ** 1.06–1.44
**Sweetened beverage consumption**			
**(Overall *p*-value)**	(<0.001)	0.004	0.018
Daily	1	1	1
Weekly	0.46 ** 0.31–0.68	0.61 * 0.42–0.89	0.87 0.62–1.23
Monthly	0.40 ** 0.27–0.59	0.51 ** 0.35–0.74	1.15 0.82–1.62
Less than monthly or none	0.44 ** 0.30–0.64	0.60 ** 0.42–0.87	0.97 0.70–1.34
**Water consumption**			
**(Overall *p*-value)**	0.501	0.259	0.067
Daily	1	1	1
Weekly	1.07 0.86–1.34	1.08 0.87–1.35	0.93 0.76–1.13
Monthly	0.68 0.35–1.30	0.82 0.43–1.56	0.63 0.34–1.13
Less than monthly or none	1.07 0.90–1.26	1.16 0.99–1.36	0.84 * 0.73–0.98
**Tea consumption**			
**(Overall *p*-value)**	(<0.001)	(<0.001)	(<0.001)
Daily	1	1	1
Weekly	0.68 ** 0.57–0.81	0.68 ** 0.57–0.81	1.19 * 1.02–1.39
Monthly	0.89 0.53–1.51	0.68 0.40–1.12	0.99 0.63–1.54
Less than monthly or none	0.51 ** 0.45–0.58	0.54 ** 0.48–0.61	0.53 ** 0.47–0.59
**Coffee consumption**			
**(Overall *p*-value)**	0.441	0.87	0.13
Daily	1	1	1
Weekly	0.70 0.40–1.24	0.99 0.57–1.75	1.16 0.73–1.86
Monthly	0.96 0.48–1.93	1.11 0.56–2.22	1.40 0.80–2.46
Less than monthly or none	0.73 0.45–1.20	0.93 0.57–1.52	0.94 0.63–1.42

* *p*-value < 0.05, ** *p*-value < 0.01; OR: 1.00 (Reference level).

**Table 3:** Pairwise comparisons between each beverage intake frequency from Tukey’s honest significance tests. Data source: China Health and Nutrition Survey, 2011.

Pairwise Comparisons	Smoking Experience	Current Smoking Status	Past Year Alcohol Consumption
**Sweetened beverage consumption:**			
Weekly–Daily	<0.001	0.042	0.858
Monthly–Daily	<0.001	0.002	0.845
Less than monthly or none–Daily	<0.001	0.028	0.997
Monthly–Weekly	0.452	0.277	0.011
Less than monthly or none–Weekly	0.902	1	0.504
Less than monthly or none–Monthly	0.63	0.122	0.052
**Water consumption:**			
Weekly–Daily	0.916	0.877	0.866
Monthly–Daily	0.613	0.919	0.376
Less than monthly or none–Daily	0.857	0.24	0.102
Monthly–Weekly	0.514	0.829	0.564
Less than monthly or none–Weekly	1	0.947	0.838
Less than monthly or none–Monthly	0.501	0.696	0.74
**Tea consumption:**			
Weekly–Daily	<0.001	<0.001	0.11
Monthly–Daily	0.97	0.396	1
Less than monthly or none–Daily	<0.001	<0.001	<0.001
Monthly–Weekly	0.724	1	0.835
Less than monthly or none–Weekly	0.003	0.027	<0.001
Less than monthly or none–Monthly	0.125	0.813	0.023
**Coffee consumption:**			
Weekly–Daily	0.596	1	0.912
Monthly–Daily	1	0.99	0.629
Less than monthly or none–Daily	0.591	0.989	0.992
Monthly–Weekly	0.687	0.978	0.857
Less than monthly or none–Weekly	0.992	0.971	0.363
Less than monthly or none–Monthly	0.7	0.885	0.219

Statistical significance: *p* < 0.05.
